# Fracture of the Upper Third of Femur

**Published:** 1892-02

**Authors:** 


### FRACTURE IN THE UPPER THIRD OF THE FEMUR.

Allis (*Medical News*, vol. lix., No. 21) carefully reviews the mechanism of fracture in the upper third of the femur exclusive of the neck. He apparently shows that the treatment ordinarily adopted in such cases cannot possibly produce the best results, and terminates his paper by the following conclusions: After fracture of the shaft, the fragments are bound together by a hinge. The hinge will be short if the vulnerating force was only sufficient to break the bone. If the hinge is short, overlapping can only occur to a very limited degree. In all such cases the "shortening" will be due to an angular displacement. If the vulnerating force is greater than necessary to merely fracture the bone, the unexpended force will tear the hinge freely. When the hinge is loose, overlapping, with or without angular deformity, is possible. The shorter the hinge, the greater will be the control of the upper fragment, through the agency of the lower. Traction on the lower fragment, under all circumstances, is incapable of restoring the long axis of the broken bone. If the hinge is short, traction will draw down the upper fragment, but cannot efface the anterior angular tendency. If the hinge is long, traction will exert but little influence upon the upper fragment. Traction in the oblique direction has no advantage over horizontal extension. The so-called "weakness" after fracture of the femur is not due to deficient bone-repair. "Weakness" is greatest when due to angular deformity, with rotation of fragments. Angular deformity, with rotation of fragments, compels both hip and knee to assume abnormal relations to the trunk. Shortness, due to overlapping, *without* angular deformity or rotation of fragments, is no barrier to heavy, manual labor. Treatment directed to the lower fragment is the probable agency in causing rotation in the lower fragment. No surface treatment can insure a useful limb. Osteotomy has corrected many a faulty union after months of wasted time. The convertion of a sealed (simple) fracture into an exposed (compound) one offers the only possible means for accurate diagnosis, and the only possible method of rational treatment. Patients and surgeons who stop short of this proceedure must compromise with best results.—*The Therapeutic Gazette.*

